# Mitochondrial ATP Synthesis and Proton Transport Synergistically Mitigate Oligodendrocyte Progenitor Cell Dysfunction Following Transient Middle Cerebral Artery Occlusion via the Pbx3/Dguok/Kif21b Signaling Pathway

**DOI:** 10.7150/ijms.100127

**Published:** 2024-08-13

**Authors:** Yehai Li, Min Zhang, Jinchuan Lin, Hang Guo, Hao Zhou, Yong Jin, Zhao Yang

**Affiliations:** 1 Department of Neurosurgery, The Affiliated Guangdong Second Provincial General Hospital of Jinan University, Guangdong Second Provincial General Hospital, Guangzhou, Guangdong 510317, China.; 2 School of Medicine, Southern University of Science and Technology, Shenzhen, Guangdong, China.

**Keywords:** Ischemic Brain Injury, Single-Cell Analysis, Differentially Expressed Genes, Molecular Pathways, Therapeutic Strategies

## Abstract

In the realm of this study, obtaining a comprehensive understanding of ischemic brain injury and its molecular foundations is of paramount importance. Our study delved into single-cell data analysis, with a specific focus on sub-celltypes and differentially expressed genes in the aftermath of ischemic injury. Notably, we observed a significant enrichment of the "ATP METABOLIC PROCESS" and "ATP HYDROLYSIS ACTIVITY" pathways, featuring pivotal genes such as Pbx3, Dguok, and Kif21b. A remarkable finding was the consistent upregulation of genes like Fabp7 and Bcl11a within the MCAO group, highlighting their crucial roles in regulating the pathway of mitochondrial ATP synthesis coupled proton transport. Furthermore, our network analysis unveiled pathways like "Neuron differentiation" and "T cell differentiation" as central in the regulatory processes of sub-celltypes. These findings provide valuable insights into the intricate molecular responses and regulatory mechanisms that govern brain injury. The shared differentially expressed genes among sub-celltypes emphasize their significance in orchestrating responses post-ischemic injury. Our research, viewed from the perspective of a medical researcher, contributes to the evolving understanding of the molecular landscape underlying ischemic brain injury, potentially paving the way for targeted therapeutic strategies and improved patient outcomes.

## Introduction

Acute cerebral infarction (ACI) is one of the most common cerebrovascular diseases, accounting for approximately 70% of acute cerebrovascular diseases 1, 2. It results from various causes that lead to localized disruption of blood supply in the brain, resulting in insufficient cerebral blood flow, ischemia, and hypoxia in brain tissue 3. This leads to localized necrosis and cerebral softening, ultimately causing severe impairment of brain function. Cerebral infarction has a high incidence, recurrence rate, mortality rate, and disability rate, imposing a heavy burden on society and families 1, 2. Based on previous research, during the progression of cerebral infarction, processes such as angiogenesis, blood-brain barrier disruption, and inflammatory responses interact closely, collectively affecting neuronal survival, synaptic repair, and regeneration. Rapid diagnosis and timely intervention for cerebral infarction positively impact its prognosis. In current clinical practice, imaging techniques are widely employed for the diagnosis of cerebrovascular diseases 1, 2. However, they still face drawbacks like high costs, complex procedures, and the need to move patients 4, 5. Therefore, there is a pressing need to explore more convenient and effective diagnostic and prognostic evaluation methods.

Under normal circumstances, mitochondrial mass and morphology balance are maintained through mitochondrial autophagy. In general, a certain level of mitochondrial autophagy contributes to maintaining mitochondrial quality and adaptability to different environments. However, during the process of tissue ischemia, there are significant changes in energy supply, and the role of mitochondrial autophagy in preserving mitochondrial function and structure, as well as the optimal intensity of mitochondrial autophagy, remains a subject of debate 4, 5. During cerebral hypoxia-ischemia, the disruption of brain blood flow leads to mitochondrial dysfunction, reduced ATP production, and increased production of reactive oxygen species (ROS). Energy depletion triggers a cascade of reactions, disrupting ion gradients on the neuronal and glial cell membranes, causing cell swelling and cytotoxic edema 6. Additionally, calcium overload, lipid peroxidation, and oxidative stress can lead to blood-brain barrier disruption, brain edema, and neuronal death. Once the inflammatory response cascade is activated, various cytotoxic substances, including nitric oxide (NO), matrix metalloproteinase (MMP), tumor necrosis factor-alpha (TNF-α), and interleukin-1 (IL-1), are released, leading to platelet aggregation, microvascular blockage, further cell damage, and damage to the blood-brain barrier (BBB) and extracellular matrix 6.

In recent years, the PINK1-Parkin pathway has been one of the most studied pathways in mitochondrial research. Recent studies have indicated that AFPR can significantly reduce neurological scores and infarct area in cerebral ischemia-reperfusion rats, alleviate cortical neuronal apoptosis, and increase hippocampal Nissl density. Furthermore, AFPR significantly promotes angiogenesis by increasing microvessel density, VEGFA expression, SIRT3 expression, and activating Pink1/Parkin-mediated mitochondrial autophagy 5, 7. Additionally, research by Yuan *et al.* found that extracellular signal-regulated kinase (ERK) activation, dynamin-related protein 1 (Drp1)/mitochondrial fusion protein 2 (Mfn2)-dependent mitochondrial dynamics imbalance, and excessive autophagy are involved in cerebral ischemia-reperfusion injury leading to neurologic damage after cardiac arrest/cardiopulmonary resuscitation. In SH-SY5Y cell models, ERK inhibition downregulates autophagy by reducing Drp1/Mfn2-dependent mitochondrial fission, opposing mitochondrial dysfunction, and promoting neuronal survival. Recent research also suggests that hypoxia induces mitochondrial biogenesis 5, 7. Hypoxia increases the production of peroxisome proliferator-activated receptor-gamma coactivator 1-alpha (PGC-1), downstream mitochondrial transcription factors (mitochondrial transcription factor A and nuclear respiratory factor 1), and heat shock protein 60 (HSP60) 4, 5. This groundbreaking discovery indicates that mitochondrial biogenesis is a novel endogenous neuroprotective response. Mitochondrial dysfunction and excessive oxidative stress play significant roles in ischemic cascade reactions. Recent research suggests that mitochondrial biogenesis and ROS detoxification are two important endogenous protective mechanisms in acute cerebral infarction and chronic neurodegenerative diseases. However, their specific regulatory mechanisms are not yet clear. Therefore, this study is based on single-cell suspension and transcriptome chip integrative analysis to examine the core regulatory cell subpopulations and regulators during the stages of cerebral infarction.

## Methods

### Ethical statement

This study adhered to the Declaration of Helsinki and the ethical guidelines of Fujian Medical University Union Hospital. The Ethics Committee approved the experimental protocols, with the reference number GSE208222.

### scRNA-seq analysis

In this study, we conducted a single-cell mining analysis, based on GSE208222 database^8^, ^9^, to explore the gene expression profile of oligodendrocyte progenitor cells (OPCs) isolated from the lateral ganglionic eminence of mice at postnatal day 10 (p10). The mice were subjected to transient middle cerebral artery occlusion (MCAO), and the OPCs were isolated two weeks post-MCAO. This specific time point was chosen to capture the molecular changes in OPCs during the recovery phase after ischemic insult. The methodology employed in this analysis involved the isolation of OPCs from the lateral ganglionic eminence, followed by the extraction of RNA to generate single-cell RNA-seq libraries 10. Subsequently, high-throughput sequencing was performed to obtain comprehensive gene expression profiles at the single-cell level.

The obtained data were then preprocessed to filter out low-quality cells and normalize expression levels. The intricate landscape of oligodendrocyte progenitor cells (OPCs) was unveiled through a sophisticated analytical duo—Principal Component Analysis (PCA) and t-Distributed Stochastic Neighbor Embedding (t-SNE) 10. The resulting PCA plot served as a visual representation, offering insights into individual cell relationships and unveiling latent structures. Complementing PCA, t-SNE, a non-linear dimensionality reduction technique, provided a nuanced perspective by preserving local relationships between cells. This approach, crucial for deciphering complex cellular landscapes, created visually compelling t-SNE plots. These plots accentuated distinct OPC subpopulations, capturing both global structures and finer details of OPC diversity. Together, the synergistic use of PCA and t-SNE not only transformed intricate gene expression data into accessible visualizations but also facilitated a profound exploration of OPC heterogeneity. This analytical tandem played a pivotal role in understanding how OPCs dynamically respond to stimuli such as transient middle cerebral artery occlusion (MCAO) 10.

### DEGs dection

In the pursuit of unraveling the intricate molecular landscape shaped by transient middle cerebral artery occlusion (MCAO) within oligodendrocyte progenitor cells (OPCs), a rigorous two-fold analysis was undertaken. Firstly, a detailed examination through differential expression analysis meticulously identified genes undergoing significant shifts in expression levels in response to the ischemic insult 11. This granular exploration shed light on the molecular protagonists orchestrating adaptive or pathological changes within the OPC population post-MCAO.

### Gene functional enrichment analysis

Of the Gene Ontology (GO) and Kyoto Encyclopedia of Genes and Genomes (KEGG) pathway enrichment analyses 11, 12. Starting with the preparation of input data, typically a list of differentially expressed genes (DEGs), researchers install and load the necessary Bioconductor packages, including 'clusterProfiler.' Subsequently, gene IDs are converted using annotation packages like 'org.Hs.eg.db' for human genes, and GO enrichment analysis is executed using the 'enrichGO' function. The results are then visualized through a bar plot, offering insights into significantly enriched GO terms 13. For KEGG pathway enrichment analysis, researchers employ the 'enrichKEGG' function, and the outcomes are visualized using a bubble plot. Additional analyses, such as the functional annotation chart created with the 'dotplot' function, further enhance the understanding of enriched terms 14. The final step involves interpreting the results, focusing on the most significant GO terms and KEGG pathways, thereby providing valuable insights into the functional implications of DEGs in the biological system under study 15.

### Gene Set Enrichment Analysis and Gene Set Variation Analysis

Expanding the analytical horizon, Gene Set Enrichment Analysis (GSEA) was deployed, transcending individual genes to unveil broader biological implications. GSEA scrutinized the distribution of genes within predefined sets or pathways, unraveling enriched biological processes and signaling pathways 16, 17. This holistic approach provided a comprehensive panorama of how the concerted activity of genes contributed to the functional landscape of OPCs in the aftermath of MCAO 18. The harmonized results from both the differential expression analysis and GSEA forged a multidimensional understanding of the molecular cascades triggered by MCAO in OPCs. This dual-pronged approach not only spotlighted specific genes responding to the ischemic challenge but also unveiled the intricate biological pathways and processes orchestrating the cellular response 19.

Gene Set Variation Analysis (GSVA) is a computational method for assessing biological pathway variations across samples. After organizing gene expression data, installing necessary R packages, and loading gene sets, preprocessing steps include data transformation and normalization 20. The GSVA algorithm is then applied to evaluate pathway activities, with an optional step for differential analysis using 'limma' for statistical testing 21-23. Visualization tools like heatmaps aid in understanding pathway variations. Statistical analyses may involve tests and p-value adjustments, and results can be integrated with clinical data for deeper insights. The methodology emphasizes thorough documentation for reproducibility and the generation of concise reports summarizing findings.

### Protein-Protein Interaction Networks Consruction

The STRING (Version: 12.0; https://string-db.org/) online platform serves as a user-friendly gateway for conducting in-depth analyses of protein-protein interactions (PPIs)^11^, ^24^. Upon accessing the STRING web portal, users can input protein names, gene IDs, or sequences of interest, generating a visual PPI network where protein nodes are connected by edges representing interactions. This platform allows for the customization of network parameters, including confidence score thresholds, refining the displayed interactions. Detailed information about individual proteins, such as functional annotations and known interactions, can be accessed by clicking on nodes within the network. STRING offers robust visualization tools for tailoring the network layout, color-coding, and node size, facilitating clear and interpretable representations. Researchers can explore topological features, such as node degrees and betweenness centrality, to identify hub proteins and central nodes 25. The platform also supports functional enrichment analyses, including Gene Ontology terms and pathway enrichment, offering insights into biological processes. Module detection algorithms aid in identifying cohesive protein clusters, further elucidating the modular organization of the network. Researchers can conveniently download visualized networks and tabular data for further analysis. STRING integrates experimental validation tools, allowing users to cross-reference predicted interactions with existing experimental data.

## Results

### Deciphering Post-Ischemic Brain Responses: Single-Cell RNA-Seq of Neuronal Subpopulations

**Figure 1:**
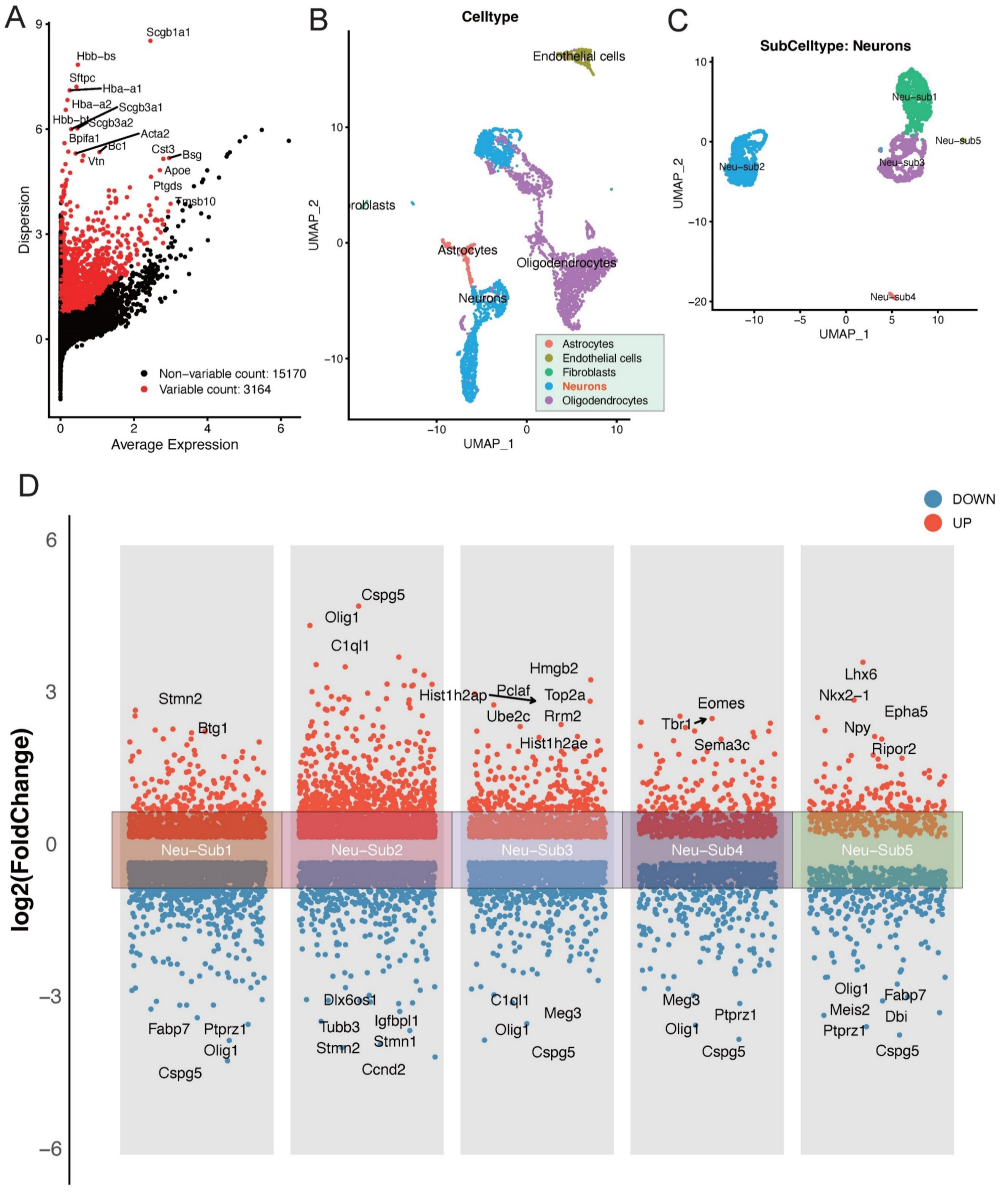
Deciphering Post-Ischemic Brain Responses: Single-Cell RNA-Seq of Neuronal Subpopulations. A highlights the primary differentially expressed genes post-ischemic injury, featuring key players such as Scgb1a1, Scgb3a2, Acta2, Bpifa1, and Bc1. B and C categorize cellular subpopulations into Astrocytes, Endothelial cells, Fibroblasts, Neurons, and Oligodendrocytes. Notably, Neurons further divide into five distinct neuronal subgroups. D provides a detailed view of the differential gene expression within these neuronal subgroups, showcasing the major upregulated and downregulated genes.

In our exploration of the single-cell dataset GSE208222, originating from murine brains post-ischemic injury, a group of pivotal differentially expressed genes has come to the forefront. Notably, genes such as Scgb1a1, Scgb3a2, Acta2, Bpifa1, and Bc1 have displayed significant alterations in their expression patterns (Figure 1A). The dataset can be broadly classified into distinct cellular subpopulations, encompassing Astrocytes, Endothelial cells, Fibroblasts, Neurons, and Oligodendrocytes (Figure 1B). Of particular interest, the Neurons subpopulation can be further subdivided into five distinct neuronal subgroups (Figure 1C). A thorough analysis of these neuronal subgroups has unveiled captivating dynamics in gene expression. Within Neu-Sub1, there is prominent upregulation of Stmn2 and Btg1, while Fabp7, Ptprz1, Cspg5, and Olig1 are among the downregulated genes. In Neu-Sub2, upregulated genes include Cspg5, Olig1, and C1ql1, with Dlx6os1, Igfbpl1, Tubb3, Stmn1, and Stmn1 notably downregulated. Neu-Sub3 exhibits upregulation in genes such as Hist1h2ap, Hmgb2, Pclaf, Top2a, Ube2c, and Rrm2, while downregulated genes comprise C1ql1, Olig1, Meg3, and Cspg5. In Neu-Sub4, genes like Tbr1, Eomes, and Sema3c are primarily upregulated, while Meg3, Olig1, Ptprz1, and Cspg5 show significant downregulation. Neu-Sub5, in contrast, demonstrates upregulation of genes including Lhx6, Nkx2-1, Epha5, Npy, and Ripor2, while Olig1, Fabp7, Meis2, Dbi, and Ptprz1 are observed in a downregulated state (Figure 1D).

### Analysis of Differential Gene Expression and Key Regulatory Pathways in Neu-Sub1 and Neu-Sub2

**Figure 2:**
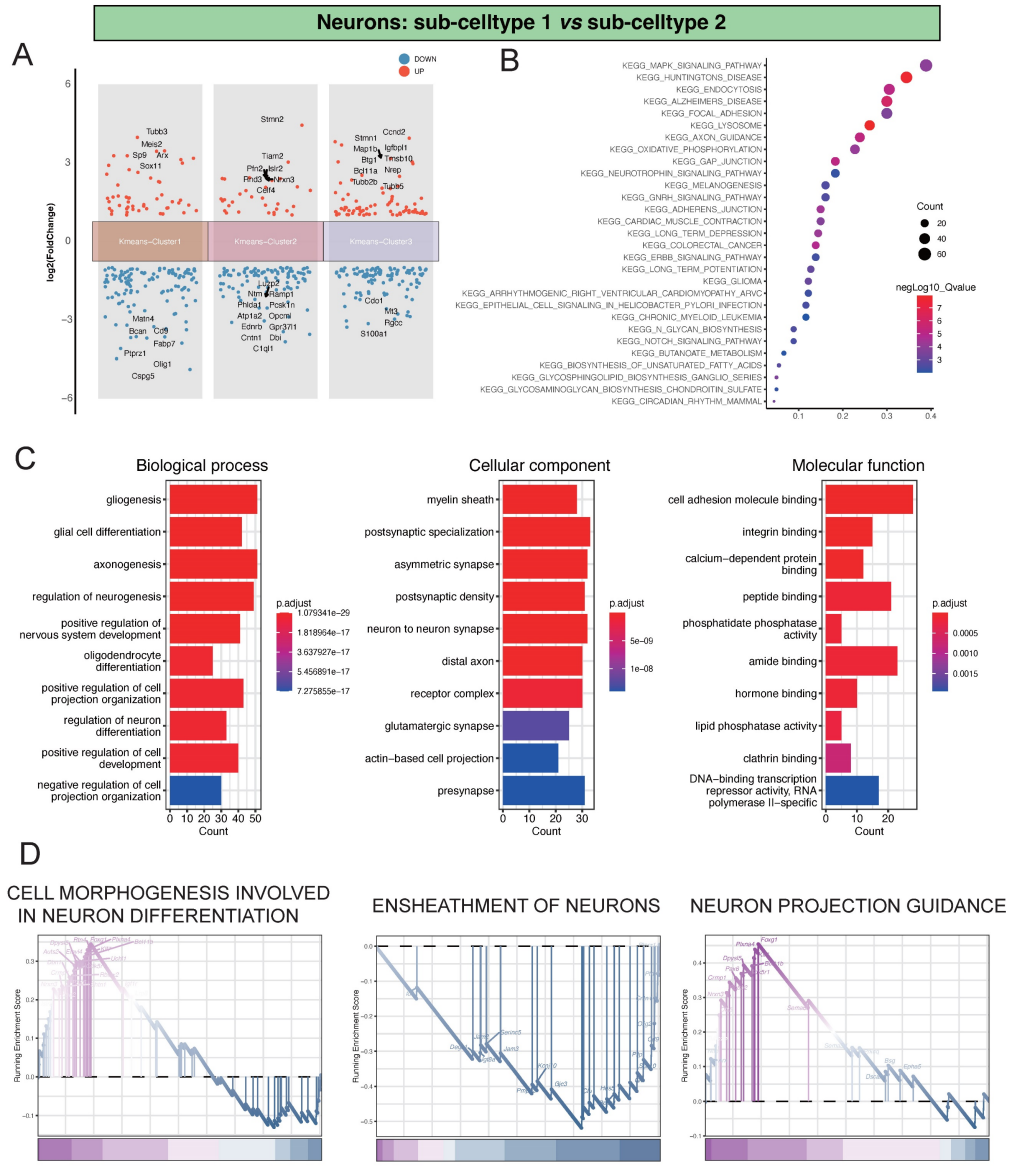
Analysis of Differential Gene Expression and Key Regulatory Pathways in Neu-Sub1 and Neu-Sub2. A showing the differential gene expression analysis between Neu-Sub1 and Neu-Sub2 reveals distinct clusters of genes distributed across three main clusters. B presenting the KEGG pathway enrichment analysis demonstrates a significant enrichment of differentially expressed genes in pathways such as KEGG_MAPK_SIGNALING_PATHWAY, KEGG_HUNTINGTONS_DISEASE, KEGG_ENDOCYTOSIS, KEGG_ALZHEIMERS_DISEASE, and KEGG_FOCAL_ADHESION. Panel C portrays the outcomes of Gene Ontology (GO) analysis, presenting enriched biological processes (BP), cellular components (CC), and molecular functions (MF) related pathways. D showing the results GSEA unveil prominent pathways associated with neuronal damage post-ischemic injury, including CELL MORPHOGENESIS INVOLVED IN NEURON DIFFERENTIATION, ENSHEATHMENT OF NEURONS, and NEURON PROJECTION GUIDANCE.

In our investigation of the single-cell dataset, focusing on the comparison between Neu-Sub1 and Neu-Sub2, we identified distinctive differential gene expression patterns grouped into three main clusters. Within Kmeans-Cluster1, we observed the upregulation of genes like Tubb3, Meis2, Sp9, Arx, and Sox11, contrasted by the downregulation of Matn4, Bcan, Cd9, Fabp7, and Ptprz1. In Kmeans-Cluster2, genes like Stmn2, Tiam2, Pfn2, Islr2, and Rnd3 were upregulated, while Luzp2, Ntm, Ramp1, Phlda1, and Pcsk1n were notably downregulated. Kmeans-Cluster3 displayed upregulation in genes such as Stmn1, Ccnd2, Map1b, Igfbpl1, and Btg1, with Cdo1, Mt3, Rgcc, and S100a1 among the downregulated genes (Figure 2A). Furthermore, KEGG pathway enrichment analysis revealed that these differentially expressed genes were primarily associated with pathways such as KEGG_MAPK_SIGNALING_PATHWAY, KEGG_HUNTINGTONS_DISEASE, KEGG_ENDOCYTOSIS, KEGG_ALZHEIMERS_DISEASE, and KEGG_FOCAL_ADHESION, providing valuable insights into the underlying biological processes and signaling pathways (Figure 2B). Additionally, GO analysis indicated significant enrichments in various biological processes (BP), including gliogenesis, glial cell differentiation, and axonogenesis. Cellular component (CC) terms such as myelin sheath, postsynaptic specialization, and asymmetric synapse exhibited substantial enrichment. Molecular function (MF) terms, encompassing cell adhesion molecule binding, integrin binding, and calcium-dependent protein binding, were also significantly enriched, highlighting the intricate molecular mechanisms at play (Figure 2C). Notably, GSEA analysis identified pivotal pathways like CELL MORPHOGENESIS INVOLVED IN NEURON DIFFERENTIATION, ENSHEATHMENT OF NEURONS, and NEURON PROJECTION GUIDANCE, shedding light on the primary processes driving neuronal damage post-ischemic injury (Figure 2D).

### Analysis of Differential Gene Expression and Key Regulatory Pathways in Neu-Sub1 and Neu-Sub3

**Figure 3:**
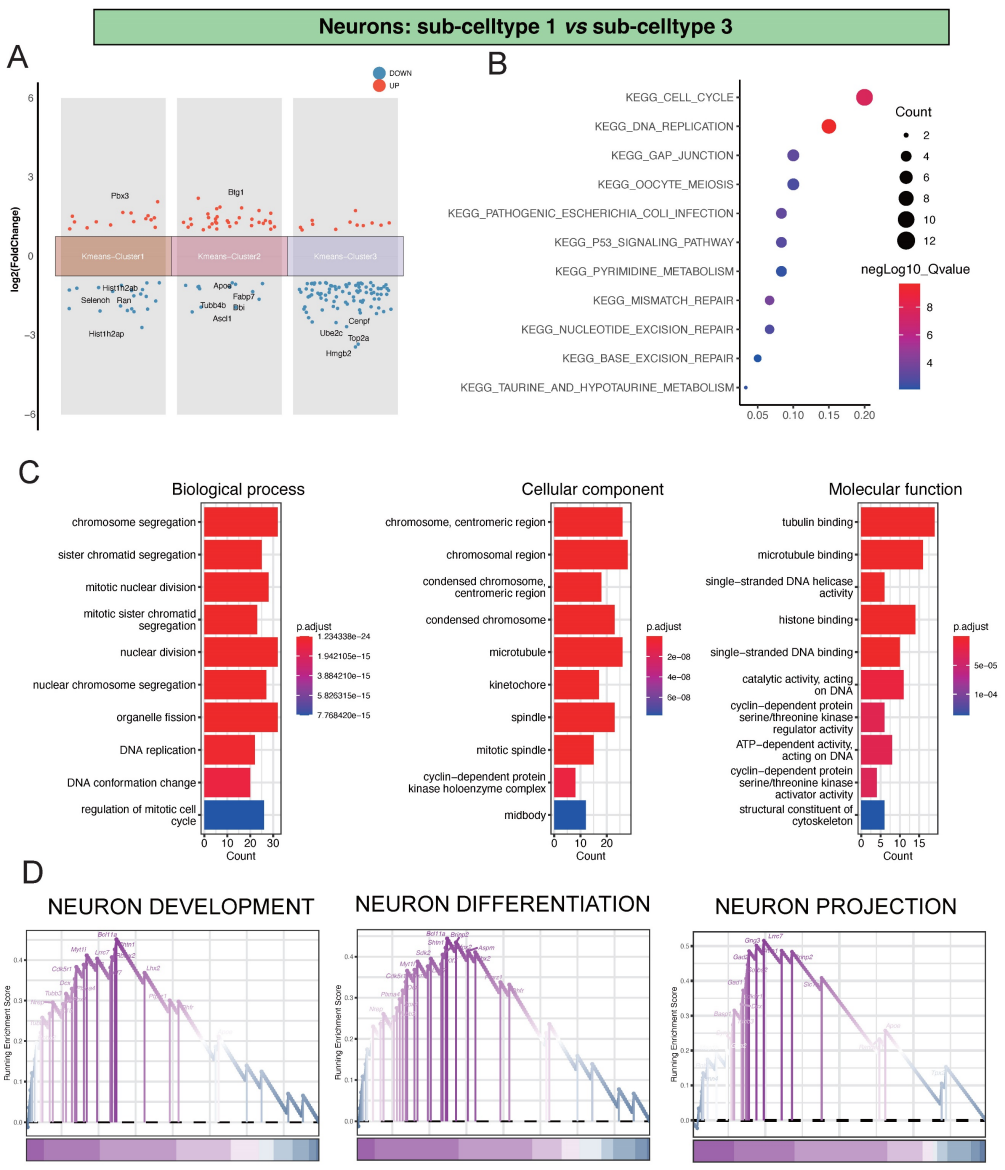
Analysis of Differential Gene Expression and Key Regulatory Pathways in Neu-Sub1 and Neu-Sub3. Panel A illustrates the outcomes of the differential gene expression analysis between Neu-Sub1 and Neu-Sub3, revealing the primary differentiation into three distinct clusters of differentially expressed genes. In Panel B, the KEGG pathway enrichment analysis showcases a predominant enrichment of differentially expressed genes in pathways such as KEGG_CELL_CYCLE, KEGG_DNA_REPLICATION, and KEGG_GAP_JUNCTION. C presents the results of Gene Ontology (GO) analysis, highlighting the enrichment of biological processes (BP), cellular components (CC), and molecular functions (MF) related pathways. The findings from GSEA are depicted in Panel D, identifying NEURON DEVELOPMENT, NEURON DIFFERENTIATION, and NEURON PROJECTION as the primary pathways associated with neuronal damage post-ischemic injury.

Regarding the comparison between Neu-Sub1 and Neu-Sub3, we unearthed significant differential gene expression patterns grouped into three primary clusters. Within Kmeans-Cluster1, we noted the upregulation of Pbx3, while Hist1h2ab, Selenoh, Ran, and Hist1h2ap showed notable downregulation. Kmeans-Cluster2 revealed an upregulation of Btg1, with Apoe, Fabp7, Tubb4b, Dbi, and Ascl1 downregulated. Kmeans-Cluster3 exhibited downregulation of genes such as Cenpf, Ube2c, Top2a, and Hmgb2(Figure 3A).

Furthermore, our KEGG pathway enrichment analysis unveiled that these differentially expressed genes were predominantly associated with pathways like KEGG_CELL_CYCLE, KEGG_DNA_REPLICATION, and KEGG_GAP_JUNCTION, providing crucial insights into the biological processes and signaling pathways at play (Figure 3B). Additionally, our GO analysis indicated substantial enrichments in various biological processes (BP), encompassing chromosome segregation, sister chromatid segregation, and mitotic nuclear division. In terms of cellular components (CC), there was significant enrichment in terms like chromosome, centromeric region, chromosomal region, and condensed chromosome, centromeric region. Molecular functions (MF) such as tubulin binding, microtubule binding, and single-stranded DNA helicase activity also showed notable enrichment, underscoring the complex molecular mechanisms involved (Figure 3C). Of particular significance, our GSEA analysis highlighted critical pathways such as NEURON DEVELOPMENT, NEURON DIFFERENTIATION, and NEURON PROJECTION, shedding light on the primary processes driving neuronal damage post-ischemic injury (Figure 3D).

### Hub markers detection and validation

**Figure 4:**
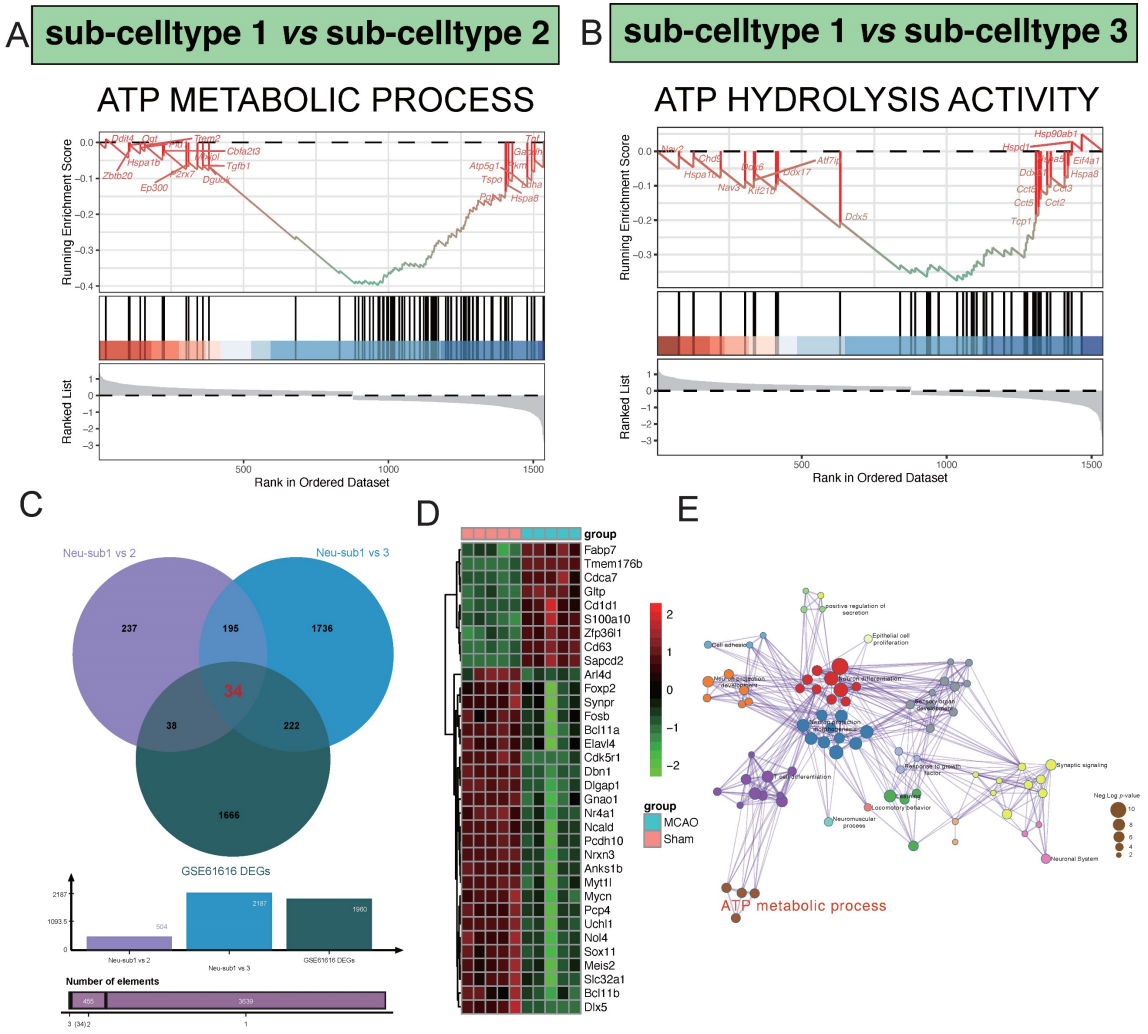
Hub pathway detection in the procesiion of post-ischemic brain responses. A highlights the significant enrichment of the ATP METABOLIC PROCESS pathway in the differential gene expression analysis between sub-celltype 1 and sub-celltype 2. In Panel B, the enrichment of ATP HYDROLYSIS ACTIVITY is notably observed in the differential gene expression analysis between sub-celltype 1 and sub-celltype 3.C demonstrates the identification of 34 common differentially expressed genes among sub-celltype 1 vs sub-celltype 2, sub-celltype 1 vs sub-celltype 3, and GSE61616 DEGs. D primarily showcases the collective expression changes of the 34 identified genes across different sub-celltype comparisons.E presents the results of pathway enrichment network analysis, revealing key pathways such as Neuron differentiation, Neuron projection morphogenesis, and T cell differentiation as central clusters actively involved in regulatory processes.

In our comparative analysis between sub-celltype 1 and sub-celltype 2, a noteworthy enrichment was observed in the "ATP METABOLIC PROCESS" pathway. Key regulatory genes within this pathway, including Dguok, Tgfb1, Pgk1, Hspa8, and P2rx7, exhibited significant differential expression (Figure 4A). Similarly, in the comparison between sub-celltype 1 and sub-celltype 3, we identified a distinct enrichment pattern in the "ATP HYDROLYSIS ACTIVITY" pathway, featuring genes such as Kif21b, Ddx17, Ddx5, Tcp1, and Cct5 (Figure 4B). By intersecting the results from sub-celltype 1 vs. sub-celltype 2, sub-celltype 1 vs. sub-celltype 3, and GSE61616 DEGs, we uncovered 34 common differentially expressed genes (Figure 4C). Within the MCAO group, notable upregulated genes included Fabp7, Tmem176b, Cdca7, Gltp, and Cd1d1, while downregulated genes encompassed Sox11, Meis2, Slc32a1, Bcl11b, and Dlx5, among others (Figure 4D). Pathway enrichment network analysis revealed key clusters of pathways involved in regulatory processes (Figure 4E)., such as "Neuron differentiation," "Neuron projection morphogenesis," "T cell differentiation," "Synaptic signaling," and "ATP metabolic process."

**Figure 5:**
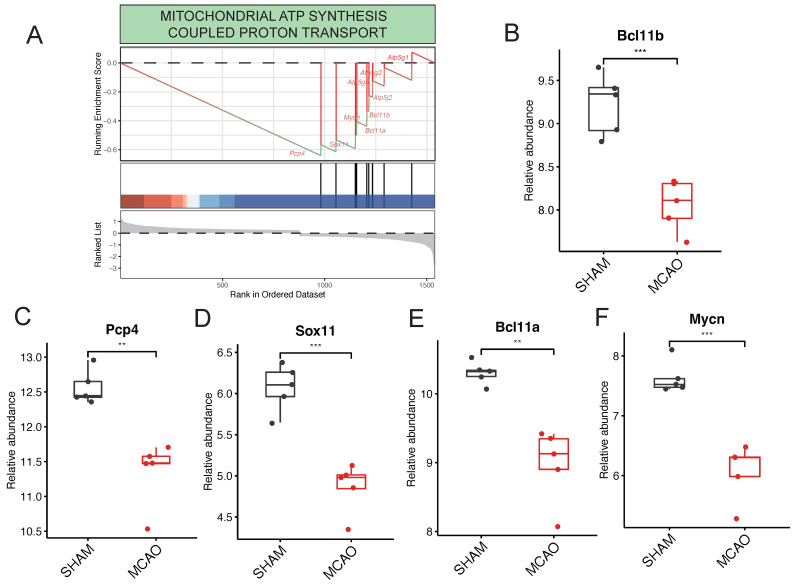
Key Regulatory Genes detection in the procesiion of post-ischemic brain responses. A highlights the pronounced enrichment of the "MITOCHONDRIAL ATP SYNTHESIS COUPLED PROTON TRANSPORT" pathway within ATP generation-related pathways. B to F collectively underscore pivotal genes, including Pcp4, Sox11, Bcl11a, Mycn, and Bcl11b, identified as major players actively participating in the regulatory processes.

Within the context of pathways related to ATP generation, it's noteworthy that the "MITOCHONDRIAL ATP SYNTHESIS COUPLED PROTON TRANSPORT" pathway stands out as significantly enriched (Figure 5A). This pathway is predominantly governed by key regulatory genes such as Pcp4, Sox11, Bcl11a, Mycn, and Bcl11b. Interestingly, in the GSE61616 gene set, these target genes consistently exhibit elevated expression levels within the MCAO group (Figure 5B-F). This consistent upregulation underscores their central role in governing the "MITOCHONDRIAL ATP SYNTHESIS COUPLED PROTON TRANSPORT" pathway.

## Discussion

Mitochondrial energy metabolism plays a pivotal role in the microenvironment of the nervous system. Numerous past studies have delved into the role of mitochondrial activity in the process of ischemic stroke, including energy sourcing and the generation of inflammatory mediators. Mitochondria primarily generate the majority of ATP through the mitochondrial respiratory chain (MRC) and oxidative phosphorylation (OXPHOS), serving as the cellular powerhouse 26. Their double-membrane structure and rich enzyme content make them central to intracellular biosynthesis. Given the high energy demands of neurons, they are more susceptible to damage and death due to mitochondrial dysfunction. Additionally, mitochondria produce large molecular precursors of metabolism, such as lipids, proteins, DNA, RNA, as well as metabolic byproducts like reactive oxygen species (ROS) and ammonia, and they have mechanisms for waste clearance or utilization. Significant changes occur in mitochondria under ischemic and hypoxic conditions, including calcium influx, mitochondrial permeability transition pore (mPTP) opening, reactive oxygen species (ROS) generation, DNA damage and mutations, mitochondrial dynamics imbalance, and mitochondrial positioning anomalies 27, 28. These changes are closely associated with patients with ischemic stroke and various animal models. Mitochondria, acting as receptors for various stimuli, trigger both caspase-independent (AIF, apoptosis-inducing factor) and caspase-dependent (cytochrome c) cell death. Ischemic and hypoxic conditions promote these processes, leading to neuronal necrosis and apoptotic death. Inflammation is another key factor in the pathophysiology of brain ischemia 28, 29. Glial cell activation, peripheral leukocyte infiltration, and damage-associated chemicals such as high-mobility group proteins, nucleotides, nucleic acid fragments, and purines trigger inflammatory responses following ischemia. Mitochondria also play a role in these inflammatory changes. Considering the vital role of mitochondria in the process of ischemic stroke, we have identified a series of differentially expressed genes involved in the development and progression of the disease through bioinformatics screening.

BCL11B, a zinc finger transcription factor, is primarily responsible for establishing proper connectivity in subcortical neurons, particularly in the fifth-layer motor neurons 30. BCL11B is also associated with the differentiation of spiny neurons in the striatum during embryonic development. Furthermore, in the adult brain, BCL11B is expressed in the basal ganglia, fifth cranial nerve nucleus, olfactory bulb, and spinal cord. Early studies found that BCL11B (B-cell CLL/lymphoma 11B) is crucial for spinal cord spiny neuron differentiation, striatal patch development, and striatal cell structure establishment. Moreover, there are reports suggesting that BCL11B serves as a novel regulatory factor in the brain-derived neurotrophic factor (BDNF) signaling pathway 30, which is disrupted in many neurological disorders. Recent research, combining expression studies with *in vivo* MRI and neural functional deficit score monitoring in individual animals with ischemic lesions, concludes that BCL11B expression is positively correlated with post-ischemic neurorecovery, indicating its beneficial role in injury repair following ischemia 31, 32. PCP4, also known as PEP-19, is a highly expressed protein in Purkinje cells. PCP4 belongs to the calmodulin family and possesses homologous calmodulin (CaM) binding domains, with anti-apoptotic and calmodulin-binding functions 33, 34. It is reported that PCP4 is also expressed in the olfactory cortex of non-human primates, though its function in these brain regions remains unclear. However, this protein is associated with the regulation of calcium and calcium-dependent signal transduction, often achieved through restricting calcium and calmodulin activity by interacting with CA2 and other calmodulins 33, 35. Here, PCP4 actively regulates neurotransmitter release and axon growth in cell lines. Additionally, PCP4 mRNA and protein levels can be regulated by steroid hormones such as estrogen and steroid hormone receptors. The PCP4 gene is located on chromosome 21, particularly in the critical region for Down syndrome (DS), suggesting a direct correlation with the pathogenesis of DS^34^, ^36^. Sox11 plays a pivotal role in regulating neuronal survival, making it a crucial regulatory factor in the development of sensory neurons and a factor in tumor stem cells 37. In the central nervous system, overexpression of Sox11 promotes neuronal maturation in developing chicken neural tubes. Sox11 is abundantly expressed during embryonic development but has very low expression levels in adult sensory ganglia (Tanabe, 2003). Following peripheral nerve injury, Sox11 mRNA is upregulated in adult dorsal root ganglia (DRG). Overexpression of Sox11 enhances excitability in dentate gyrus (DG) granule cells and reduces levels of various potassium channel subunits, indicating the critical importance of Sox11 activity in regulating the plasticity of DG neurons 38, 39. Our research findings suggest that Sox11 is crucial for neuronal survival under oxygen-glucose deprivation/reoxygenation (OGD/R) conditions and provides protection against stroke-induced damage. Furthermore, Sox11 mediates neuroprotection by minocycline under OGD/R conditions. BCL11A belongs to a protein transcription factor family containing C2H2 zinc finger domains and is highly expressed in the central nervous system 32, 40, 41. It is indispensable for neural development and has been found to be upregulated at early time points after nerve injury. Lack of BCL11A in neurons of the central nervous system leads to differentiation interruption and reduced free radical migration, thereby affecting morphogenesis and neural innervation. Reports suggest that in the central nervous system, BCL11 controls polarity and migration of upper-layer neurons during neocortical development. Studies have demonstrated that BCL11A may mediate Schwann cell activation and peripheral nerve regeneration by binding to the promoter of nuclear receptor subfamily 2 group F member 2 (Nrf2) 32, 40, 41. Thus, BCL11A is crucial for Schwann cell activation and peripheral nerve regeneration. MYCN is a member of the MYC proto-oncogene family, which also includes MYC and MYCL, encoding a basic helix-loop-helix-leucine zipper (bHLH-LZ) transcription factor 42, 43. Overexpression of the MYCN gene is a characteristic feature of many embryonal brain tumors, leading to enhanced cell proliferation and cell cycle disruption. Early studies suggest that overexpression of MYCN in neuroblastoma cells results in a transcriptome rich in typical MYC target genes, including those involved in ribosome biogenesis and protein synthesis 43, 44. MYCN functions as a transcriptional regulator of many target genes involved in critical cellular processes, including but not limited to metabolism. A functional gene set characteristic of MYCN has been identified in a neuroblastoma cell line, suggesting that MYCN suppresses genes related to neuronal differentiation 43, 45.

In summary, our current research has preliminarily confirmed the critical roles of core genes, including Bcl11b, Pcp4, Sox11, Bcl11a, and Mycn, in ischemic stroke disease. Further extensive research is required to validate these findings and potentially provide new insights for the treatment of ischemic stroke.
